# Attractiveness and determinants of maxillary midline diastemas in a West African smile

**DOI:** 10.1038/s41405-025-00302-2

**Published:** 2025-02-18

**Authors:** Sefaakor Ahiaku, Brian J. Millar, Michael Cronin

**Affiliations:** 1 Distance Learning Postgraduate Mentor & Facilitator, Faculty of Dentistry, Oral and Craniofacial Sciences, King’s College London, London, UK; 2 Associate Dentist, Northway Dental Practice, Liverpool, UK; 3 Associate Dentist, Orchard Dental Practice, Wirral, UK; 4 Clinical Professor of Dental Education, Faculty of Dentistry, Oral and Craniofacial Sciences, King’s College London, London, UK; 5 NHS Consultant in Restorative Dentistry, King’s College London, London, UK; 6 Senior Lecturer, Discipline of Statistics, School of Mathematical Sciences, University College Cork, Cork, Ireland

**Keywords:** Restorative dentistry, Fixed prosthodontics, Aesthetic dentistry, Fixed prosthodontics, Dental treatments

## Abstract

**Introduction:**

This cross-sectional study examines whether the assumption that diastemas of 1 mm or greater are un-attractive is true in a Black West-African population, and identifies the determinants of attractiveness.

**Methods:**

A structured questionnaire was self-administered to adult participants, who rated digitally altered full-face colour photographs of a male and female model with diastema widths of 0–6 mm on a 5-point Likert scale. Quantitative data was analysed using SAS® (version 9.4) and SPSS® (version 26). Qualitative data was analysed with thematic analysis.

**Results:**

375 participants completed the questionnaire (51% female, 48% male, 1% preferred not to disclose) with a modal age of 25–34. 65% of participants found maxillary midline diastema esthetic and/or desirable, with 63% of those with diastemas choosing to have no treatment, and the desire to have treatment decreasing with increasing age (*p* = 0.012). No smile was perceived to be unattractive, however female smiles were rated more favourably (*p* < 0.001) and increasing diastema width correlated with a decreasing attractiveness especially for diastemas wider than 3 mm in females and 2 mm in males. Diastema width, the gender of the model, and whether or not the participant had a diastema themselves was shown to impact perceptions of attractiveness in a multi-variable analysis.

**Conclusion:**

There is limited evidence to support the position that MMDs over 1 mm are not attractive in this population. Diastemas of ≤3 mm in females and ≤2 mm in males are considered attractive. In addition gender, diastema width, and an individual’s own diastema (or absence thereof) impact perceived attractiveness.

## Introduction

Perceptions of Maxillary Midline Diastemas (MMD) vary significantly across the continents and cultures of the world, however dental textbooks almost universally suggest that they should be eliminated or reduced to 1 mm or less in order to achieve an attractive smile [1]. Despite there being notable exceptions to this in the media, esthetic dental trends still support the elimination of the midline diastema where possible. The MMD however, has recently experienced a renaissance in the modelling world, in part, due to evolving beauty standards [2, 3].

Much of the existing research into MMDs is based within predominantly Caucasian populations, however, most of this research concedes that little consideration has been given to populations that are non-Caucasian. In West Africa, perceptions about midline diastemas and spacing in general differ significantly from those often expressed in the literature, with a gap often being revered [4] and a sign of good future fortune amongst some groups. In light of this, diastemas of 4 mm or less are commonly accepted in West Africa [5].

In West Africa, like the Western world, there has been an increased interest and availability of cosmetic dental treatments available, with some individuals being willing to travel abroad to obtain treatment. The pursuit of Western standards of beauty [6] as a result of European and American [7] influence have in part contributed to this increase.

### The dentist’s role

As dentists, it is important that we consider cultural and racial factors as they pertain to our patients. This understanding could help inform our treatment plans and ensure our patients are making informed decisions. Providing patient-specific and culturally appropriate care requires the clinician to be aware of cultural norms and our own natural inclinations in prescribing treatments, to ensure we do not impose a smile design strictly fashioned on a Western beauty standard.

## Aims

The aim of this study was to examine perceptions about MMDs and its determinants in a specifically Black West-African population, in order to understand how the existing perceptions in that population compare with the published evidence about MMD acceptability.

### Objectives

1. Ascertain the desirability of MMD in this population.
2. Compare these findings against the published research in this area.

## Materials and methods

**Fig. 1:**
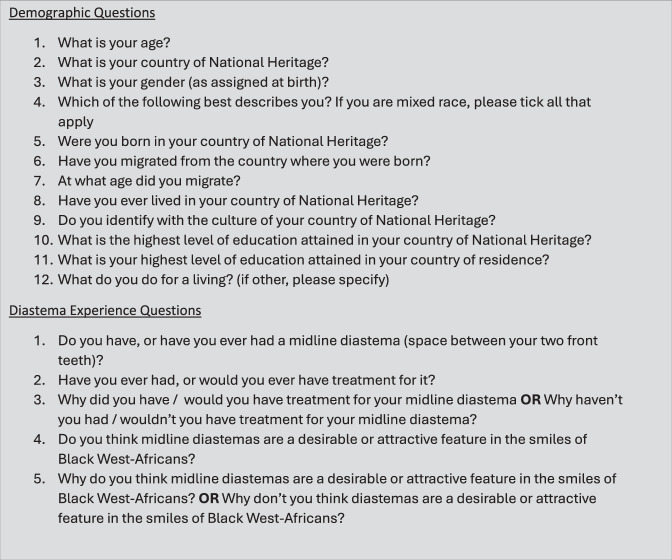
Questionnaire administered to participants.

**Fig. 2:**
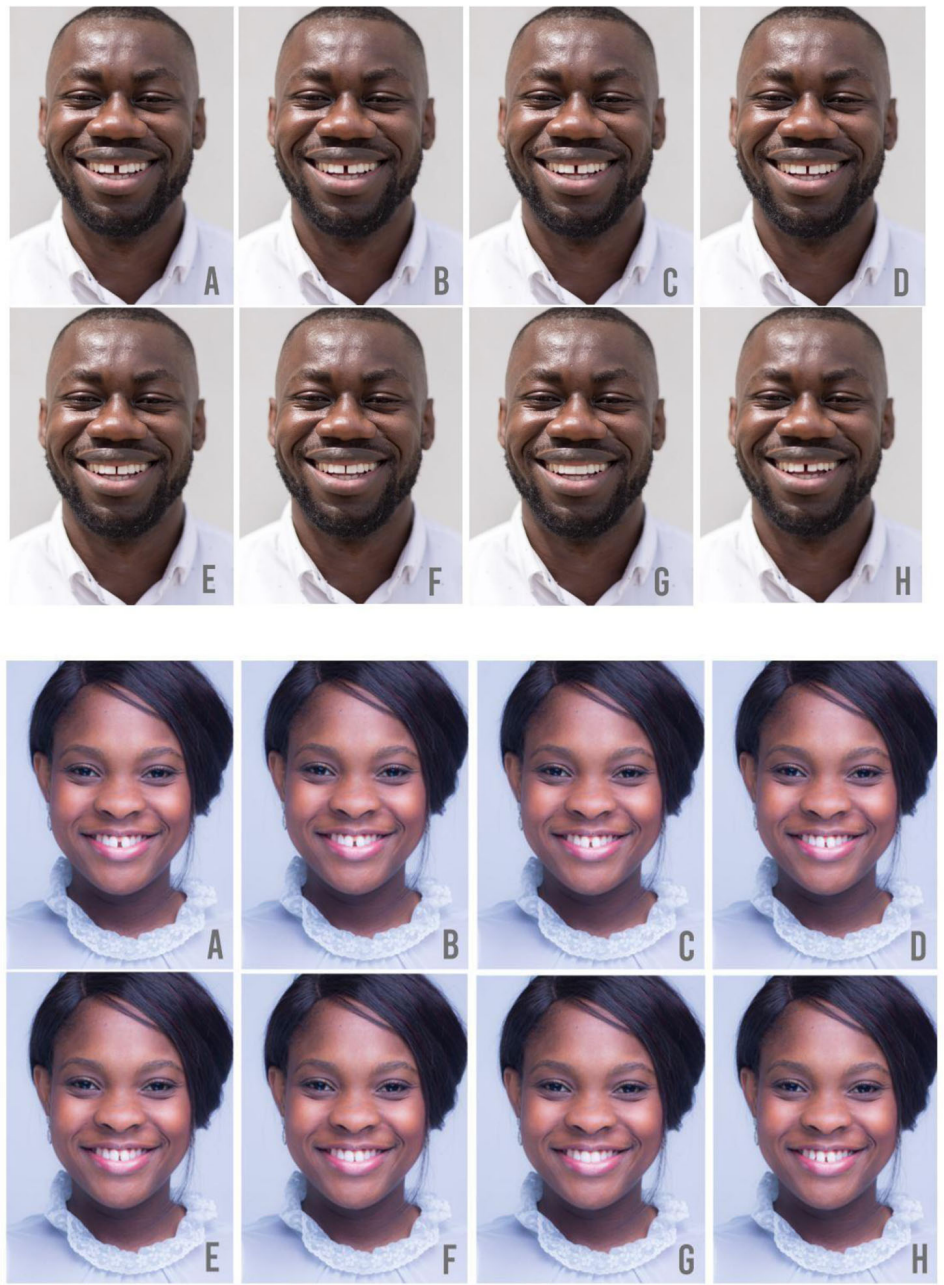
Simulated Male & Female Smiles. Simulated male/female smiles with the following diastema sizes: **A** – 6 mm, **B** – 5 mm, **C** – 4 mm, **D** – 3 mm, **E** – 2 mm, **F** – 1 mm, **G** – 0 mm, **H** – original photo.

A structured self-administered questionnaire (Fig. 1) was administered asking participants a series of questions about themselves, their experience of maxillary midline diastemas, and their opinions of 8 female and 8 male full-face photographs (Fig. 2).

The 16 photographs represented simulated smiles with a 0–6 mm diastema for each model. To obtain the simulated smiles informed consent was obtained from two models aged 25–35 known to one researcher who were subsequently recruited. Both models had Ghanaian heritage (of at least 2 generations), had an existing diastema and had not undergone any orthodontic or cosmetic dental treatment to change the shape or the size of their teeth. Both models had however undertaken a course of tooth whitening. A suitable image of each model was taken using a DSLR camera and this image was altered to create the simulated smiles in Adobe Photoshop (version: Creative Cloud 2019). The images were first harmonised by creating dental symmetry across the midline, thus removing distracting features in each smile. This produced ‘male smile C’ (4 mm) and ‘female smile D’ (3 mm) as the harmonised images of each model’s existing smile. The central incisors were then edited to make the diastema smaller (by widening the central incisors) or larger (by narrowing the central incisors). No other parameters were adjusted in each image. Participants were asked to rate the desirability and attractiveness of the 16 smiles on a 5 point Likert scale, with 1 representing a very attractive smile, 5 representing a very unattractive smile, and 3 representing an acceptable smile.

The questionnaire format and design were formulated following a literature review of some existing papers from around the world [1, 8–16]. A combination of quantitative and qualitative data was collected. Within the questionnaire, the order in which the 16 images appeared was randomised for each participant.

The questionnaire was administered using the web-based tool TypeForm™ between 3^rd^ March 2021 and 12^th^ April 2021 at participants’ convenience on their own device. The study was based online, and as such participants were not included or excluded based upon their geographical location, enabling global participation. An opportunistic sampling method was used to contact potential participants via gatekeepers (community leaders and network members known to one researcher within the target population). Each participant was subsequently invited to share the link to the survey with any potential participants they knew. Participants in the questionnaire were all aged 18 or older and had heritage in West-Africa.

### Power calculations

A minimum participant number of 271 was determined. This sample size calculation was based on estimating the proportion that would rate MMDs as being attractive to within 5 percentage points with 95% confidence, without any prior assumption of the value of the proportion.

### Statistical methods

A preliminary analysis of the data was conducted using SPSS® (version 26) to provide summary statistics and Chi Squared tests. The qualitative data was analysed using thematic analysis.

For further analysis, the scale of the attractiveness score was reversed before statistical analyses for ease of interpretation, so that higher scores represented higher perceptions of attractiveness. The demographic variables were screened individually for inclusion in the analysis (Table 1).

**Table 1:** Demographics and questionnaire responses of participants and means (and standard deviation (SD)) of attractiveness scores.

Variable	Category	*n*	(%)	Mean attractiveness score (SD)
Age	18–24	51	14%	3.8 (1.0)
	25–34	116	31%	3.7 (1.0)
	35–44	75	20%	3.7 (1.0)
	45–54	33	9%	3.7 (1.0)
	55–64	61	16%	3.9 (0.9)
	65+	39	10%	3.6 (1.0)
	Total	375	100%	3.7 (1.0)
Country of national heritage	Ghana	263	70%	3.7 (1.0)
	Nigeria	106	28%	3.7 (1.0)
	Other	6	2%	3.9 (1.1)
Gender (as assigned at birth)	Female	192	51%	3.7 (1.0)
	Male	180	48%	3.7 (0.9)
	Prefer not to say	3	1%	3.3 (1.0)
Born in country of national heritage	Yes	317	85%	3.7 (1.0)
	No	58	15%	3.7 (1.1)
Migrated from country of birth	Yes – away from country of national heritage	163	43%	3.7 (1.0)
	Yes – back to or close to country of national heritage	24	6%	3.7 (0.9)
	No	188	50%	3.7 (1.0)
Lived in country of national heritage	Yes – 5 years or more	323	86%	3.8 (1.0)
	Yes – between 3 months and 5 years	18	5%	3.6 (1.1)
	No	34	9%	3.6 (1.1)
Identify with the culture of country of national heritage	Yes	355	95%	3.7 (1.0)
	Not sure	14	4%	3.5 (1.1)
	No	6	2%	3.5 (0.9)
Education level	Second level	16	4%	3.6 (1.0)
	Undergraduate degree	125	33%	3.8 (1.0)
	Diploma/masters/postgraduate degree	181	48%	3.7 (1.0)
	Doctorate	51	14%	3.8 (1.0)
	Prefer not to say	2	1%	4.4 (0.6)
Have/had a midline diastema	Yes & would not/not sure they would have treatment	115	31%	3.9 (0.9)
	Yes & did/would have treatment	43	11%	3.5 (1.0)
	No/not sure	217	58%	3.7 (1.0)

The variables were individually screened using a linear mixed model and were retained for the next step of the analysis if significant at the 10% level. The retained variables were then included together in a multi-variable model and backward elimination was used to retain only those variables significant at the 5% level. All possible 2-way interactions were added to the model and backward elimination was used to retain only those interactions significant at the 5% level of significance in a final model. The participants were included in the models as a random effect. Diagnostics were performed on the residuals to confirm the suitability of the final model. Statistical analyses were performed using SAS® (version 9.4).

## Results

There was a total of 375 participants in the survey, all of whom were included in the statistical analyses. There was a reasonably even spread of ages among the participants, with slightly higher representation of the 25–34 years age group (31%) and slightly lower representation of the 45–54 years age group (9%) (Table 1). The majority of participants were of Ghanian national heritage (70%), followed by Nigerian national heritage (28%) and other West African countries (2%). There was an even spread of gender (as assigned at birth), with 51% female, 48% male and 1% preferring not to say. The majority of participants were born in their country of national heritage (85%), had lived there for at least 5 years (86%) and identified with the culture of that country (95%). Half (50%) of the participants had not migrated from their country of birth, 43% had migrated away from their country of national heritage and 6% had migrated back to or close to their country of national heritage. One third (33%) of participants had an undergraduate degree, nearly half (50%) had a postgraduate degree and 14% had a doctorate.

**Fig. 3:**
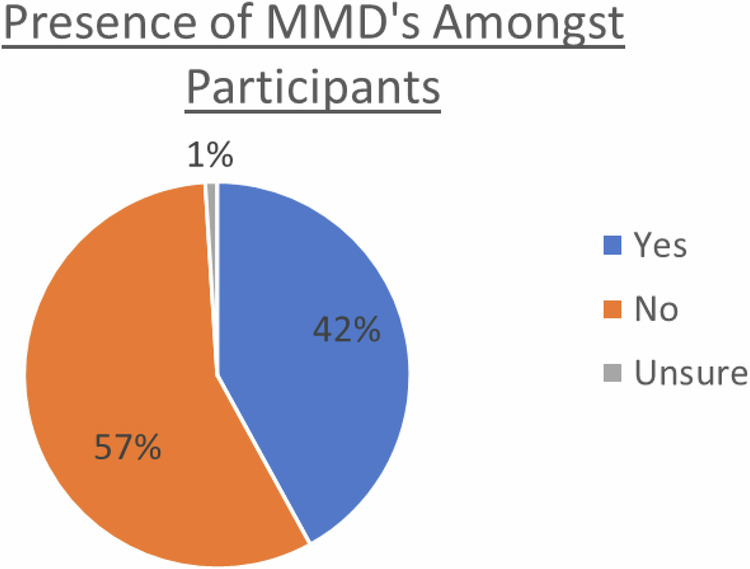
Incidence of maxillary midline diastema amongst participants.

**Fig. 4:**
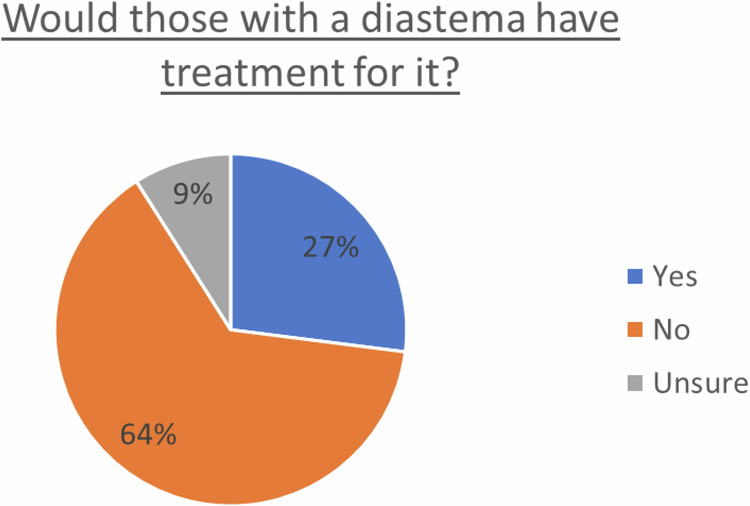
Likelihood of seeking treatment for MMD.

Over half (58%) of participants did not have (*n* = 212) or were not sure (*n* = 5) they had a midline diastema (Fig. 3). Almost one third (31%) did have a midline diastema and either would not have or were not sure they would have treatment for it (Fig. 4). One in nine (11%) did have a midline diastema and either already had or would have treatment for it. Overall, 18% of males and 25% of females had a diastema. Desire to treat an existing diastema decreased with age (*p* = 0.012).

### Desirability of MMDs

**Fig. 5:**
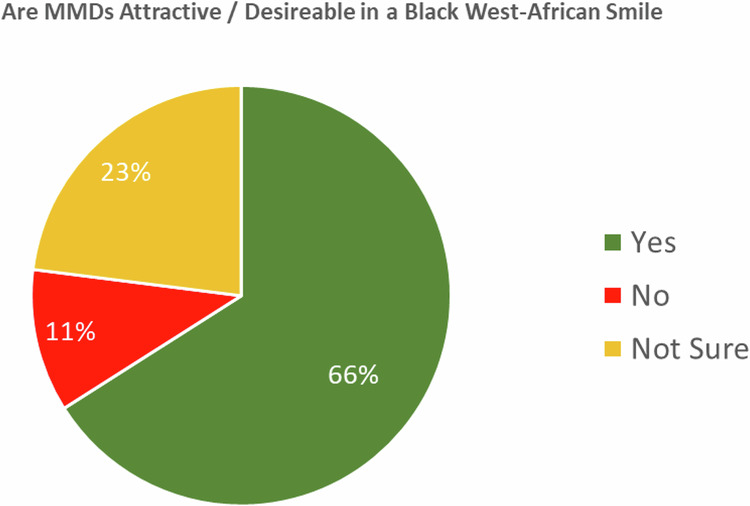
Perceived attractiveness of MMDs.

Positive views of MMDs were held by 246 (66%) participants, 42 (11%) held negative views and 87 (23%) were unsure (Fig. 5). The proportion of participants who found MMDs attractive was found to be equal to the average of the figures (70%) found in the existing research (*p* = 0.057) [5, 7–9, 17–20].

### Interactions between variables

The following variables were found to be un-associated with perception of attractiveness in the individual variable screening models: country of national heritage (*p* = 0.5240) and migrated from country of birth (*p* = 0.3639).

The following variables were found to be un-associated with perception of attractiveness in the multi-variable model and were removed from the model: age (*p* = 0.4287), gender (0.5441), born in country of national heritage (*p* = 0.8315), lived in the country of national heritage (*p* = 0.2087), identifying with the culture of the country of national heritage (*p* = 0.1629) and education level (*p* = 0.0732). Thus the following variables were associated with perception of attractiveness and were retained in the final model: having a midline diastema combined with ever having treatment for it, gender of the subject in the photograph and the width of the midline diastema in the photograph. Each of the two-way interactions between these variables were significant and were also retained in the final model (Table 2).

**Table 2:** Final linear mixed model analysis of perception of attractiveness.

Variable	Num DF	Den DF	*F* value	*p*-value
Have/had a midline diastema	2	4868	1.27	0.2810
Gender of photo	1	4868	177.86	<0.0001
Width of diastema	1	4868	575.27	<0.0001
Have/had a midline diastema * gender of photo	2	4868	7.10	0.0008
Have/had a midline diastema * width of diastema	2	4868	20.79	<0.0001
Gender of photo * width of diastema	1	4868	19.29	<0.0001

*Num DF* Numerator Degrees of Freedom, *Den DF* Denominator Degrees of Freedom.

While each of the three variables in the final model influenced the perception of attractiveness, the presence of the interactions demonstrates that their impact is complex, as the nature of their influence depends on the other variables.

**Fig. 6:**
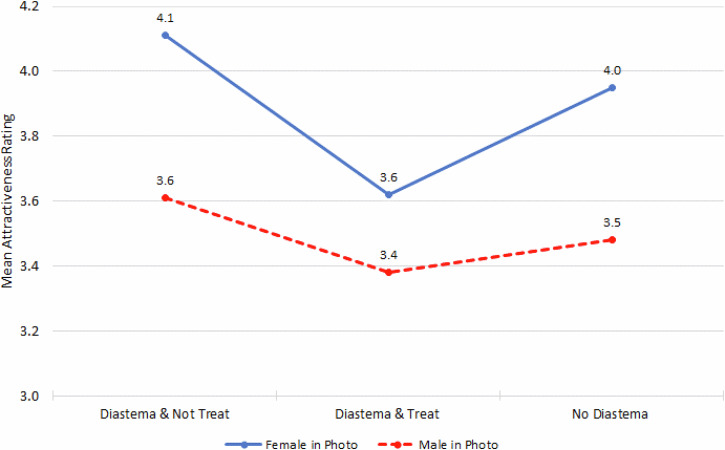
Mean attractiveness rating by have/had a midline diastema and gender in the photograph.

The influence of the participant having a diastema depended on the gender in the photograph being rated (*p* = 0.0008). Those that had a diastema and either would not have or were not sure they would have treatment for it and those that did not have or were not sure they had a diastema had a higher perception of attractiveness, particularly if the photographs they were rating were of the female rather than the male. Those that had a diastema and either already had or would have treatment for it had a lower perception of attractiveness, but the gender of the photograph was not as great a discriminant factor for them (Fig. 6).

**Fig. 7:**
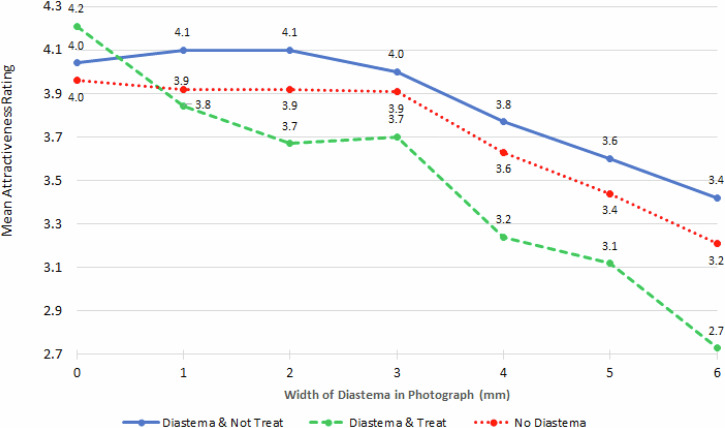
Mean attractiveness rating by have/had a midline diastema and width of the diastema in the photograph.

The influence of the participant having a diastema depended also on the width of the diastema in the photograph being rated (*p* < 0.0001). Those that had a diastema and either would not have or were not sure they would have treatment for it and those that did not have or were not sure they had a diastema had a higher perception of attractiveness, except when there was no diastema (width = 0 mm), with the attractiveness decreasing similarly as the diastema in the photographs became wider. Those that had a diastema and either did already have or would have treatment for it had a lower perception of attractiveness, also with the attractiveness decreasing, but to a greater degree as the diastema in the photographs became wider (Fig. 7).

**Fig. 8:**
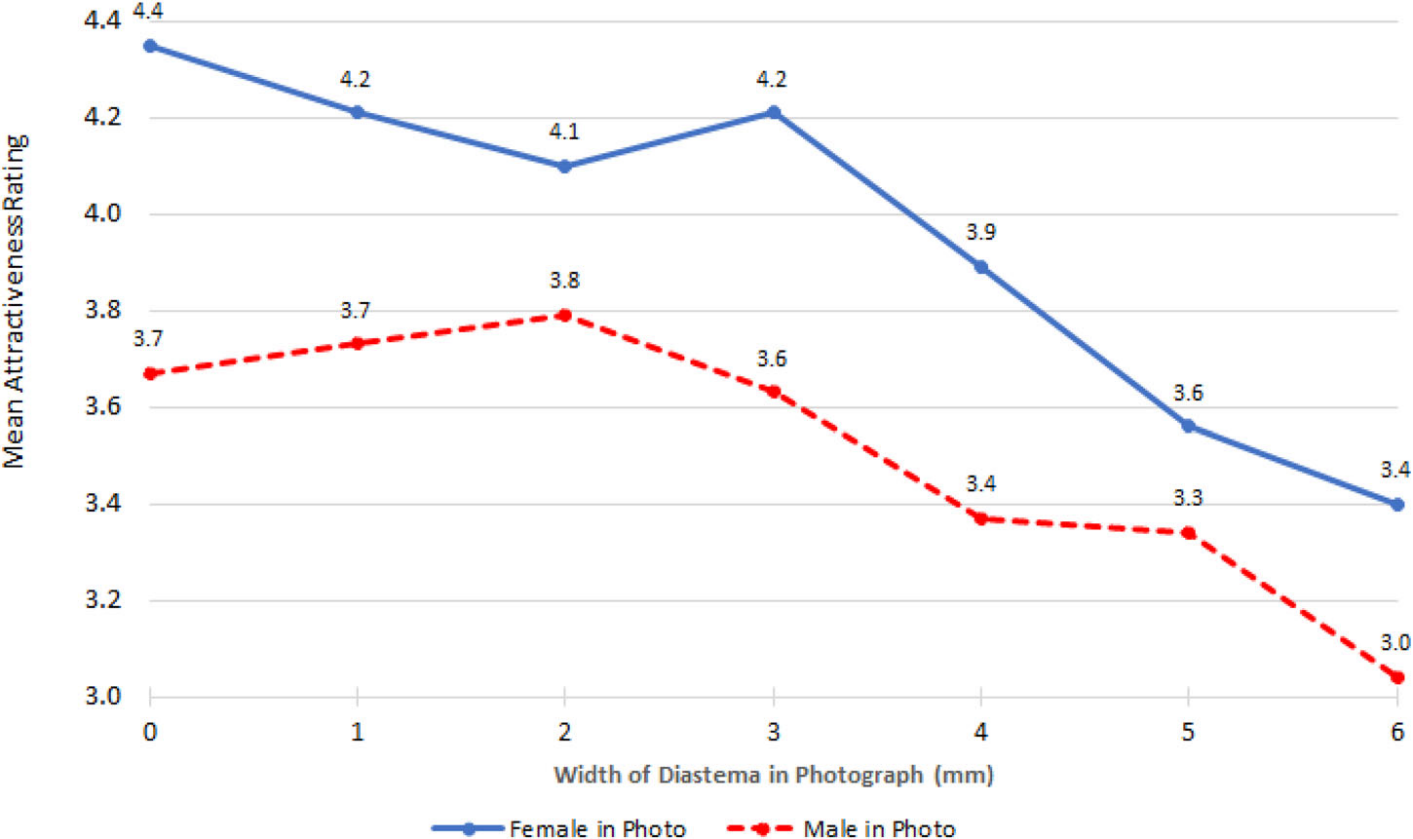
Mean attractiveness rating by gender in the photograph and width of the diastema in the photo.

The influence of the gender in the photograph being rated depended on the width of the diastema in the photograph (*p* < 0.0001). There was a higher perception of attractiveness when the photographs being rated were of a female rather than a male, with the attractiveness for the female generally decreasing as the diastema in the photographs became wider. When the photographs being rated were of the male, there was an initial increase in attractiveness up to a diastema width of 2 mm, with a reducing attractiveness thereafter (Fig. 8).

## Discussion

### Region and representation

The majority of participants came from Ghana and Nigeria, meaning that this data can only confidently relate to this portion of West-Africa. The sample population showed reasonable representation across the demographic variables, with skew in the categories place of birth, education & profession.

Given the small percentage of participants who had been born outside of their country of heritage, it can be assumed that the majority of those who now live abroad were first generation immigrants. Given the varied ages at the point of immigration, the impact of Western influence cannot be ascertained from this sample.

### Prevalence

The prevalence of Maxillary Midline Diastemas was significantly higher in this study (42%) than it was in studies of the same population included in a recent literature review [21] (17–30%). It is also slightly higher than the highest figure previously reported in the literature reported by Mabiaku and Ibawoh [17] at 36%. Given the sampling method, participants who had a diastema may have been more likely to participate resulting in a sample bias.

### Diastema width

The ideal diastema width in this study has been noted to be 0–2 mm for males and 0–3 mm for females, based upon the inflection point noted across all demographic variables. This supports the conclusions drawn from Ahiaku and Millar’s [21] literature review of 2–4 mm being the acceptable diastema width [9].

### Beauty

Based on this study, it is apparent that MMDs are more acceptable in females than they are in males, regardless of their size, with females being more accepting of diastemas, with one participant commenting “*A little diastema is desirable, even sexy, in a female smile, but less so in males*.”

The majority of participants found MMDs attractive in general. This is further supported by the mean attractiveness ratings for each smile, particularly showing that male diastemas of 1 and 2 mm are more attractive than those of 0 mm.

### Critique of methodology and suggestions

This study scored 20/22 on the Witt & Flores-Mir [22] criteria (Table 3). This criterion, in combination with a literature review [21], was used to design the study to ensure its validity.

**Table 3:** Flores-Mir methodological score for study.

Participant number	Sampling method	Presentation of photos	Viewing protocol	Intra-examiner reliability	Scoring technique	Methodological score
375 (334 lay persons)	Opportunistic Sample using gatekeepers and social media sharing INCLUSION CRITERIA • Adult aged 18+ • Heritage in West Africa	Full face photos with altered diastema widths only	Participants viewed photos one at a time in a randomly allocated order (female photos first then male photos) and could technically go back to review photos on browser	Participants subsequently viewed all photos simultaneously and rated the most and least attractive allowing the figures to be compared to independent ratings	5-point Likert Scale	20/22
4/4	3/3	6/6	3/4	1/2	3/3	

#### Sampling

Improving the representativeness of the sample would improve the quality of this data. Using a stratified-random sample, would provide results which were more generalisable. Opportunistic sampling and word of mouth is likely to have led to clusters of participants, introducing bias. Although present, this bias appears to be minimal as the trend in perception appears to be reproduced across the demographic stratifications of the data.

Hosting the questionnaire on a web-based platform, although making it easier to manage for the researcher, is likely to have excluded some sectors of the population who are either online but suspicious of the internet, or not online at all. This would have contributed to the lower numbers of participants in some portions of the population like the elderly and those in lower socio-economic categories.

#### Smile simulations

In this study, smile simulations were created by adding to the width of the central incisor, which itself is a determinant of attractiveness. The smile simulations could have been created in a number of ways, as shown by Reis et al. [1] who showed both orthodontically managed spaces and spaces closed by restorative means. All the simulations were based on restorative management alone. It is possible that simulations with orthodontic management, rather than restorative management, would have affected the perception of some of the smiles as the height : width ratio of the tooth itself would remain un-affected.

A further study could create the smile menu simulating both techniques. In addition, creating a smile simulation for a male and female from each participant’s specific country of national heritage would generate more robust data given that overall facial features do vary in the region.

Finally, some participants commented that they found no problem with any of the smile simulations but were unable to indicate that they did not find any smiles un-attractive. Should this study be repeated, allowing participants to select such an option would be beneficial.

## Conclusion

Perceived diastema attractiveness has a complex relationship with diastema width, whether or not an individual has or has not had a diastema themselves, and the gender of the person with the diastema. They are a desirable feature in the smiles of this population, and therefore it should be held as an integral factor to consider in smile design in this group. Given their perceived enhancement of beauty in parts of this population, this is a feature dentists should consider carefully before altering or eliminating it.

## Data Availability

The data supporting this article is openly available from the King’s College London research data repository, KORDS, at 10.18742/28193192.
